# The Human–Canine Partnership in Animal-Assisted Crisis Response: A Welfare-Centered Perspective

**DOI:** 10.3390/ani16182857

**Published:** 2026-09-11

**Authors:** Batya G. Jaffe

**Affiliations:** 1 Wurzweiler School of Social Work, Yeshiva University, 2495 Amsterdam Ave, New York, NY 10033, USA; jaffebatya@gmail.com; 2 G.I.L.I Lab, Geha Mental Health Medical Center, Helsinki 1, Petah Tikva 49100, Israel

**Keywords:** animal-assisted crisis response, human–canine partnership, human–animal interaction, canine welfare, crisis intervention, animal-assisted interventions

## Abstract

Animal-Assisted Crisis Response (AACR) is a specialized form of crisis intervention in which trained handlers and therapy dogs provide emotional support following crises and disasters. Although previous research has examined AACR, less is known about the contribution of the human–canine partnership. This study explored the human–canine partnership and its perceived role in effective crisis response and canine welfare. This qualitative study used semi-structured interviews and demographic surveys. Twenty-one crisis responders participated, and interview data were analyzed using Charmaz’s grounded theory approach. Findings suggest that participants perceived a highly trained handler, a carefully selected dog, and the quality of their partnership as important components of effective AACR practice. Canine welfare emerged as a fundamental component of the human–canine partnership, with participants emphasizing the handler’s role in recognizing canine stress cues, safeguarding welfare, and cultivating a strong partnership with the dog. Although bringing a dog to a crisis scene presents practical challenges, participants also described benefits for the team and improved engagement with trauma survivors. These findings support conceptualizing AACR as a welfare-centered human–canine partnership in which handler expertise, canine suitability, and attention to welfare are perceived as important components of effective and ethical crisis-response practice.

## Simple Summary

Animal-assisted crisis response depends on a close partnership between trained handlers and their dogs, who work together to provide emotional support following crises and disasters. Although previous research has examined handlers, therapy dogs, and canine welfare, less is known about how the human–canine partnership itself contributes to effective crisis response. This study explored the experiences of crisis responders to better understand this human–canine partnership. The findings suggest that participants perceived a strong partnership between a skilled handler and a carefully selected dog, with animal welfare forming an important foundation of that relationship. Handlers play a vital role in recognizing stress, protecting the dog’s physical and emotional well-being, and ensuring the dog is suited to this demanding work. Viewing AACR as a welfare-centered human–canine partnership has important implications for dog selection, handler training, welfare protocols, and the future development of crisis response programs.

## 1. Introduction

Animal-Assisted Crisis Response (AACR) is an animal-assisted intervention in which trained handlers and therapy dogs work together to provide emotional support following crises and disasters [1]. Crises and disasters are often sudden, disruptive events that can overwhelm individual and community coping resources [2]. Exposure to traumatic events may result in subsequent mental health consequences such as post-traumatic stress disorder (PTSD), anxiety, depression, and other mental health conditions [3].

Mental health crisis interventions offer emotional stabilization and psychological first aid in the aftermath of crises and disasters. This type of response aims to promote emotional stabilization and mitigate later mental health consequences for trauma survivors [4]. As a specialized form of crisis intervention, AACR provides emotional support at crisis scenes through an experienced handler and a therapy dog trained specifically for this intervention [1].

AACR is derived from the broader field of Animal-Assisted Interventions (AAI). Existing literature indicates that animal-assisted interventions may facilitate engagement and emotional stabilization among trauma-exposed individuals and communities [5,6,7,8]. Assistance and therapy animals have increasingly been incorporated into mental health settings because of their ability to facilitate therapeutic engagement and emotional support [9]. Systematic review and meta-analytic evidence further support the use of animal-assisted interventions for trauma-exposed populations [7,8]. AACR teams have become increasingly recognized as part of crisis response [10].

Emerging evidence suggests that animal-assisted therapy may enhance trauma recovery by fostering emotional safety, trust, and engagement [11]. Prior literature has explored AACR dog characteristics, handler expertise, and canine welfare [6,12,13]. Although these domains have been considered separately, less is known about how the human–canine partnership itself contributes to intervention effectiveness and canine welfare.

Dogs have long shared a close relationship with humans and have provided meaningful companionship throughout history [14]. Physiological research further suggests that positive human–dog interactions are associated with neurophysiological changes linked to bonding and stress reduction [15]. Oxytocin has been identified as one potential mechanism underlying these interactions, with evidence suggesting that human–dog contact may promote attachment, reduce stress reactivity, and contribute to emotional well-being [16]. In addition, studies have demonstrated that humans and dogs exhibit behavioral synchronization during shared activities, reflecting coordinated patterns of movement, communication, and social referencing that may further strengthen the human–canine partnership [17,18]. This close human–animal bond has also been incorporated into therapeutic and crisis intervention settings and serves as the foundation for AACR. AACR is a form of crisis intervention implemented following crises and disasters by providing emotional support and facilitating engagement with survivors and responders [19]. It consists of two members: a trained AACR handler and an AACR therapy dog who respond together in the aftermath of traumatic events [6]. During crises and disasters, AACR may offer forms of emotional support and engagement that complement conventional crisis response approaches [10,20].

Literature suggests that during times of crisis, humans and dogs seek one another for mutual support [12]. Physical contact and close proximity between humans and animals may promote stress reduction and recovery from trauma [21]. Moreover, dogs may possess a unique capacity to mediate interactions, facilitating engagement and communication [6].

Human–animal relationships are culturally and socially situated, and dogs may not be experienced as comforting, safe, or appropriate by all individuals or communities. Cultural and religious beliefs, previous experiences with animals, animal-related trauma, and community norms may influence attitudes toward dogs and the acceptability of animal-assisted interventions [22,23]. Accordingly, culturally responsive AACR practice requires consideration of recipient and community perspectives rather than assuming that interaction with dogs is universally desirable.

### 1.1. AACR Dog Characteristics

AACR dogs fulfill specific roles during crisis response. To perform these effectively, they should possess attributes that make them suitable for this type of intervention [12].

Firstly, AACR dogs should be healthy, as this may contribute directly to their resilience and ability to work properly during AACR interventions [5,6]. The nature of AACR work may involve transportation, loud noises, varied environments, crowds, long hours, and disrupted schedules; therefore, the dog’s goodness of fit should be carefully assessed [5,6]. The temperament of the dog is also imperative [8]; they should be able to remain calm through chaotic situations, tolerate stress, and respond consistently to commands [21,24].

Research identifies traits such as friendliness and affiliation toward people as critical for AACR [13]. Moreover, they should feel comfortable with people of all ages and ethnicities that may be encountered in crisis scenes. They should also be accustomed to working around other animals, as additional animals may be present at the crisis scene [6].

AACR dogs should receive specialized preparation for transportation, confinement, crowding, unfamiliar environments, and unusual sights, sounds, and smells. They should also be able to remain calm, respond reliably to commands, tolerate frequent handling and bathing, and wear protective equipment such as booties when necessary [6,21,25]. Furthermore, the training should involve obediently interacting with unfamiliar individuals [26].

### 1.2. Handler Expertise

AACR handlers should be suitable for performing this type of intervention. Traits such as compassion, sympathy, empathy, and strong listening skills are critical for handlers [13]. Handlers should also be able to communicate effectively with other crisis responders and adapt to changing conditions at the scene [6].

Handlers should undergo training to perform this intervention professionally. Most AACR organizations provide online and in-person experiential training [13]. Additional training may include Psychological First Aid (PFA) or Critical Incident Stress Management (CISM) [6].

An essential element of the AACR handler’s role is to advocate and care for their canine partner. Handlers should continuously monitor their dogs for behavioral and physiological indicators of stress, fatigue, discomfort, and declining well-being [5,19,24]. Beyond common canine stress signals, such as shaking, panting, lip licking and restlessness [6], AACR dogs may show additional signals and handlers should know their dog well enough to recognize additional signals that may include refusing to wear the work vest, turning their heads to avoid interaction with strangers, or lying down behind the handler [19].

AACR handlers should be able to recognize and respond to their dog’s stress, fatigue and burnout by establishing appropriate boundaries and promoting the animal’s well-being [21].

### 1.3. Human–Canine Partnership

AACR is delivered by a handler–dog team, and the quality of the relationship between the two may influence how they function together during crisis response. Their bond is defined as a mutually beneficial and dynamic relationship, influenced by their interactions, feelings, and behaviors associated with health and well-being [27]. Together, they are able to enter chaotic crisis scenes, relying on their mutual trust as a source of strength while caring for one another and ensuring each other’s safety and well-being [19].

The attunement and attachment between the handler and dog may provide further insight into the benefits of human–canine interaction during times of crisis [12]. Recent evidence also suggests that humans and animals may exhibit physiological co-modulation during shared interactions, indicating that coordinated emotional and physiological responses can emerge within established human–animal partnerships [15]. AACR handlers and their dogs may notice subtle changes in affect, mood and behavior. Their attunement may even involve the mirroring of experiences [19].

AACR handlers describe how their dogs helped them remain emotionally stable while providing AACR interventions. Even when experiencing grief themselves, handlers reported that their dogs helped them carry that emotional burden, enabling them to continue supporting clients [28].

### 1.4. Canine Welfare

AACR dogs may be exposed to stressful or chaotic conditions during deployment, including unfamiliar environments, novel sensory stimuli, crowds, disrupted routines, and heightened emotional intensity [19].

Emerging evidence suggests that the close human–dog relationship also involves emotional and physiological co-regulation. While this connection may buffer stress and promote resilience, it may also increase the potential for dogs to be affected by the emotional state of their handlers, highlighting the importance of safeguarding canine welfare during AACR interventions [29].

In order for dogs to perform their role successfully, both their physical and emotional health are of utmost importance [6,27,30]. Firstly, the safety of the dog at all times is critical [19,24]. To help manage the dog’s stress, frequent breaks should be provided. The dog’s physical needs should be assessed continuously, including breaks for naps, water, and walks [6]. Proactive planning of stress-relief and stress-prevention strategies for AACR dogs is essential to the effectiveness of the intervention [25].

Although existing literature highlights AACR dog characteristics, handler expertise, and canine welfare, the human–canine partnership itself remains underexplored. Understanding how crisis responders perceive the role of this partnership in AACR practice and canine welfare is an important next step in AACR research.

This study aimed to explore the human–canine partnership within AACR and examine how crisis responders perceive its role in AACR practice and canine welfare. The research question guiding this study was: “How does the human–canine partnership shape Animal-Assisted Crisis Response?” The findings contribute to conceptualizing AACR as a welfare-centered human–canine partnership.

## 2. Materials and Methods

### 2.1. Study Design

This study presents a focused secondary analysis of qualitative data originally collected as part of the author’s doctoral dissertation on Animal-Assisted Crisis Response in Israel [31]. The original doctoral study examined AACR broadly, whereas the present study revisited the original interview data to address a distinct and more focused research question concerning the human–canine partnership and canine welfare within AACR. No new participant data were collected for the present study. Demographic surveys and semi-structured interviews served as the original sources of data.

The present analysis drew on the original ATLAS.ti coding (version 24.2.1 [32295]; ATLAS.ti Scientific Software Development GmbH, Berlin, Germany) developed during the doctoral research while returning to the interview data for focused analysis of concepts relevant to the present research question. Charmaz’s constructivist grounded theory approach guided the analysis [32], allowing concepts and themes relevant to the focused research question to be developed inductively from participants’ accounts.

### 2.2. Participants and Recruitment

Participants were recruited through purposive sampling based on their direct experience responding to crisis scenes. Recruitment targeted individuals with direct operational and clinical experience responding to crisis events. Data were collected in November 2021.

The final sample consisted of 21 crisis responders representing AACR, mental health, supervisory, and other crisis-response roles. Participant roles and experience are summarized in Table 1.

### 2.3. Data Collection

A semi-structured interview guide was used to ensure consistency across interviews while allowing participants to expand on topics they considered meaningful. Interviews lasted approximately 45–60 min. Interviews were conducted in person, via videoconferencing, or in writing, according to participant preference.

The semi-structured interview guide, including the Participant Demographic Questionnaire, is provided in Supplementary Material S1.

### 2.4. Data Analysis

For the present focused analysis, the author returned to the original interview transcripts and the ATLAS.ti coding developed during the doctoral research. Existing codes relevant to the present research question were reviewed alongside the original interview data and further examined in relation to the human–canine partnership and canine welfare. The analysis retained an inductive orientation, with particular attention to participants’ language and perspectives. Codes and excerpts relevant to the focused research question were compared across interviews and organized into increasingly abstract categories and themes. Constant comparison was used to clarify relationships among emerging concepts. Analysis continued iteratively until the thematic structure adequately represented the patterns identified across the dataset.

Researcher journaling was maintained throughout data collection and analysis to enhance reflexivity, document analytic decisions, and support interpretation of the findings.

### 2.5. Ethical Considerations

Prior to the commencement of data collection, the study received an exempt determination from WCG IRB under 45 CFR § 46.104(d)(2) (WCG IRB Work Order No. 1-1491812-1). All participants provided informed consent before participation. Participation was voluntary, and measures were taken to protect participant anonymity and confidentiality throughout the study. Participants were informed that they could withdraw from the study at any time without penalty. As a token of appreciation, each participant received a restaurant voucher.

## 3. Results

### 3.1. Characteristics of AACR Dogs

Participants consistently described the characteristics of AACR dogs as important to the functioning of the human–canine partnership. AACR dogs were expected to remain calm throughout crisis scenes. A dog being too energetic or active when approaching a crisis scene can be overwhelming for trauma survivors rather than serve as a beneficial presence. The dog should feel comfortable being approached and hugged by strangers. Participants emphasized that dogs should not become anxious in response to crisis scenes or unfamiliar people. Given the unpredictable nature of traumatic scenes, participants remarked that dogs should be “safe or calm enough for that type of entrance into a scene”.

Participants described AACR dogs as appearing to identify individuals in distress and independently approaching them, thereby facilitating engagement. A participant shares:

Lucy wound her way through a very large group and ended up with some woman who I personally would not have picked out of the crowd as ‘in need,’ but Lucy somehow identified something about her that brought her over there. I don’t know if it’s smell or non-verbal or facial expression, body language; somehow Lucy is able to pick out of a crowd faster and easier in some cases, I think, than a human trauma therapist.

Participants emphasized that AACR dogs should be highly trained and carefully selected based on temperament. Unlike many other therapy animals, dogs offer the practical advantage of being able to move alongside the handler throughout a crisis scene while simultaneously providing a comforting physical presence through petting and close interaction. As one participant noted: “*...it needs to be a well-trained dog... You need to know your animals very well... A dog is not only good just because it can be hugged and petted but you can also run with it.*”

Beyond the characteristics of AACR dogs, respondents also described the reciprocal nature of the human–canine partnership.

### 3.2. Reciprocal Human–Canine Partnership

Bringing an AACR dog to a crisis scene presents both benefits and additional responsibilities for the handler. AACR handlers described how attending to the dog’s needs may increase their stress levels, as they must simultaneously care for the animal’s well-being while responding to an already tense situation. As one participant explained: “*There certainly can be challenges. One is the extra added element that you have to care for the animal as well. That takes some thought and limits the responder’s availability.*”

Participants described AACR dogs as providing handlers with a sense of calm and emotional support: “*it affects me in a very positive way because I am a dog lover, and the fact that the dog is present calms—it definitely has a positive effect on me that the dog is present.*” Similar to the mechanisms through which AACR may benefit trauma survivors, handlers described holding and petting their dogs as a form of emotional regulation and self-care.

Beyond the interaction itself, caring for the dogs after leaving the scene may also help handlers lower their own stress levels and regain a sense of control, as one respondent explained: “*Dealing with practical and technical things like maintaining the petting zoo, is a way to feel in control and to feel competent and it also has calming elements.*”

AACR handlers described feeling that they had a partner accompanying them to the crisis scene, reducing the sense of facing these complex situations alone: “*It’s providing the feeling that I have a partner and I’m not alone. It’s one of the strongest feelings you feel... When I’m with an animal, I feel as though I’m with another being.*”

While the partnership offered substantial benefits, respondents also emphasized the importance of maintaining the dog’s welfare.

### 3.3. Welfare-Centered Practice

AACR handlers emphasized that attending to the dog’s needs is imperative when responding to crisis scenes: “*You can’t ever forget the needs of the animal. And you can’t ever say* “*not now*” *to the animal.*” This requires that the animal’s welfare remain a constant consideration, even in the midst of a crisis.

An important consideration when bringing an AACR dog to a crisis scene is its safety. Crisis responders follow protocols and guidelines for their own safety; handlers must also consider the animal’s safety when attending a crisis:

Just like there’s ‘safety’ for the medic; there needs to be ‘safety’ for the dog. If I’m bringing him to an area where I know there are other dogs around, or to a fire scene where I don’t want the dog breathing in the fumes, then the same way there is safety for us, there must be safety for the animal.

It is the AACR handler’s responsibility to manage each specific crisis scene in a way that protects the dog’s well-being: “*Additionally, when I come to an event, and I know there will be many kids there who will rush to him- that’s another consideration I must make- how to figure that out.*” For example, this may involve remaining in one location and allowing people to approach the dog rather than moving through large crowds, thereby reducing unnecessary stress and discomfort. 

Attention to the AACR dog’s well-being emerged as a defining component of the human–canine partnership. Handlers should be attentive to setting boundaries and managing work time to ensure that dogs receive adequate breaks and rest and are not overworked: “I needed to watch them and establish boundaries and when it’s enough and when they needed rest. And be very attentive to their wellbeing to not exhaust them and then get to unpleasant situations.”

Caring for the dog also involves considering the frequency of attendance at crisis scenes and the individual needs of each dog to avoid overexposure: “When you work with just one dog, there’s the conflict of how much it is exposed, and how much does it affect them. There is a whole protocol in the Delta Society about this, and dogs are different from each other, how does each take it.”

The commitment to protecting the dog’s well-being was closely intertwined with the reciprocal partnership that handlers described developing with their AACR dogs.

### 3.4. Enhancing Animal-Assisted Crisis Response

Participants consistently described AACR as the product of a coordinated human–canine partnership rather than the presence of a therapy dog alone. One respondent illustrated this relationship by describing the mutual understanding and trust that had developed between him and his AACR dog: “*it’s such a gentle and man-loving breed and I calm him down and pet him and when he looks at me, I really feel as though he trusts me. We mostly have an understanding between us.*”

The visible bond between handler and dog facilitates survivors’ trust and engagement: “*Patients see how I’m worried about the animals and take care of them… and in their opinion I’m a trustworthy individual. Because they identify with the animals, this is something which really opens up.*”

AACR not only supports the delivery of psychological first aid but is also perceived as providing an additional element beyond formal protocols. According to the participants, AACR dogs may facilitate emotional comfort while also creating opportunities for engagement that are difficult to achieve through human interaction alone: “*An animal can change the atmosphere, lower people’s guards, put a smile on their face, and bring this innocent, lovable, unconditional positive regard. A dog is always going to do something different that a human couldn’t do*.”

Having the AACR dog at the scene provides people with an opportunity to approach responders without initially intending to seek emotional support: “*People naturally approach the group, no matter who is holding the leash, and start up conversations… often I don’t have to seek people out because they are seeking out the animal.*”

AACR dogs were perceived as facilitating engagement with survivors who had been difficult to engage through conventional crisis response efforts: “*People open up and become more receptive and more cooperative and find themselves speaking much more.*”

## 4. Discussion

Participants’ accounts suggest that effective AACR practice involves not simply the presence of a therapy dog but the quality of the welfare-centered human–canine dyad. This aligns with previous work highlighting the importance of the handler–dog partnership and mutual trust [12,16]. However, the present study extends this literature by suggesting that the partnership is not merely beneficial but appears to represent an important component of how participants understand effective AACR practice. Furthermore, participants described reciprocal support within the dyad as beneficial both to the intervention and to the resilience and well-being of the team itself [15].

Consistent with earlier research, participants emphasized the importance of the dog’s appropriateness for this type of intervention [5,13]. AACR dogs should undergo careful assessment and selection based on temperament, behavior, and suitability for crisis work [6]. Firstly, dogs should possess a calm, stable temperament and appear to enjoy this type of work. Secondly, they should be well-trained. The present findings suggest that these two parameters, together with the handler’s close relationship with the dog, contribute to the AACR dog’s suitability for this work.

Although participants did not explicitly describe handler expertise as a separate construct, it emerged as a critical component of successful AACR. Effective handlers require expertise in trauma-informed care and psychological first aid, together with a thorough understanding of canine behavior and welfare. Participants emphasized the importance of recognizing individual canine stress cues, directing interactions appropriately, making sound clinical judgments, and balancing operational demands with safeguarding the dog’s welfare. These findings align with previous literature emphasizing the importance of handlers being attuned to their dogs [12].

The present findings align with prior studies suggesting that a critical component of the partnership and the provision of AACR intervention is the handler’s responsibility to maintain the dog’s welfare at a high standard. The present findings suggest that welfare is not merely an ethical responsibility but was perceived by participants as an important condition for maintaining the dog’s capacity to participate in AACR and for supporting the human–canine partnership. This aligns with the literature that emphasized the importance of advocacy and welfare for the canine member of AACR [24].

Findings suggest that AACR presents both challenges and benefits for handlers. While caring for the dog introduces additional responsibilities during crisis response, participants also described the dog as a source of emotional support, calmness, and companionship. These accounts suggest that reciprocal benefits may support handler resilience and contribute to participants’ perceptions of effective AACR practice [15].

Participants perceived that some dogs could identify individuals in distress, consistent with previous literature [25]. This study extends the literature by suggesting that dogs may identify individuals in distress whom handlers might not otherwise approach, indicating that the human–canine partnership has the potential to augment crisis response and facilitate engagement.

The existing literature discusses dog selection, welfare, and the handler–dog relationship separately, whereas the present findings suggest that participants experience these elements as interconnected within a welfare-centered human–canine dyad. Therefore, the findings support conceptualizing AACR as a welfare-centered human–canine partnership in which handler expertise, canine capabilities, and welfare are perceived as important components of effective AACR practice.

The perceived benefits of AACR identified in this study should not be interpreted as indicating that dogs are universally experienced as comforting or appropriate. Human–animal relationships are shaped by cultural, religious, historical, and individual factors, and some individuals or communities may experience dogs with discomfort, fear, or avoidance [22,23]. Accordingly, the potential value of AACR should be considered within the cultural and social context of the individuals and communities being served. Cultural responsiveness and community acceptability should therefore be regarded as important considerations in determining whether and how an AACR team is deployed.

### 4.1. Limitations

This study has several limitations. The findings are based on a purposive sample of 21 crisis responders and reflect participants’ experiences and perceptions rather than direct observations of AACR practice. As a qualitative study, the findings are intended to provide in-depth insight rather than statistical generalizability. Future research using observational, mixed-methods, or longitudinal designs could further evaluate the mechanisms identified in this study. Cultural differences in attitudes toward dogs and the acceptability of AACR were not a focus of the present analysis. Accordingly, the findings should not be generalized as suggesting that dogs are universally experienced as comforting or culturally appropriate.

### 4.2. Practice Implications

The findings have several practical implications for AACR organizations. Handlers should receive comprehensive training in crisis response, psychological first aid, canine behavior, and welfare. Dogs should undergo careful assessment and screening to determine their individual suitability for AACR. Welfare protocols should remain central throughout deployment, ensuring adequate rest, monitoring for stress, and appropriate workload management. Finally, organizations should recognize the handler–dog partnership, rather than the dog alone, as the operational unit of AACR. AACR organizations should also consider cultural appropriateness and recipient and community preferences when determining whether dog-assisted crisis response is suitable for a particular setting or population.

### 4.3. Future Research

Future research should investigate the mechanisms underlying dogs’ apparent ability to identify individuals in distress, including whether dogs respond to olfactory, behavioral, physiological, or other cues when approaching them.

Future research should explore the factors that contribute to an effective human–canine partnership in AACR, including trust, communication, handler expertise, and welfare-centered practices.

Future research should also examine the experiences of trauma survivors receiving AACR to further understand how the human–canine partnership influences engagement and recovery. Recipient and community perspectives should be examined across diverse cultural, religious, and social contexts to better understand how attitudes toward dogs may influence the acceptability and perceived value of AACR.

## 5. Conclusions

This study explored the human–canine partnership within the AACR team and its perceived role in AACR practice. Findings suggest that participants perceived welfare as a fundamental component of the human–canine partnership and closely connected to the functioning of the dyad. Taken together, these findings support conceptualizing AACR not as the presence of a therapy dog, but as a welfare-centered partnership between a highly trained handler and a well-suited dog, whose combined expertise and relationship were perceived as important to effective and ethical AACR practice.

Recognizing the handler–dog partnership as the operational unit of AACR has important implications for practice, emphasizing the need for careful dog selection, comprehensive handler training, and welfare-centered protocols. By positioning the welfare-centered human–canine partnership at the center of AACR, this study provides a framework to guide the continued development, implementation, and evaluation of animal-assisted crisis response programs.

## Figures and Tables

**Table 1:** Participant Characteristics (*N* = 21).

Characteristic	*n* (%) or Mean (SD) [Range]
**Responder role**	
AACR responder	5 (23.8)
Mental health crisis-response supervisor	6 (28.6)
Mental health crisis responder	4 (19.0)
Crisis-response supervisor without a mental health background	2 (9.5)
Crisis responder without a mental health background	4 (19.0)
**Experience**	
Years of crisis-response experience	8.4 (6.7) [3,4,5,6,7,8,9,10,11,12,13,14,15,16,17,18,19,20,21,22,23,24,25]
Years working with AACR	5.7 (5.0) [3,4,5,6,7,8,9,10,11,12,13,14,15,16,17,18,19,20,21]

Note: AACR = Animal-Assisted Crisis Response. Values for responder roles are *n* (%); experience variables are Mean (SD) [Range].

## Data Availability

The data presented in this study are not publicly available due to ethical and privacy restrictions associated with qualitative interview data. De-identified data may be made available by the author upon reasonable request and subject to applicable ethical requirements.

## Supplementary Materials

The following supporting information can be downloaded at: https://www.mdpi.com/article/10.3390/ani16182857/s1, Supplementary Material S1: Semi-Structured Interview Guide (including the Participant Demographic Questionnaire).

## Abbreviations

The following abbreviations are used in this manuscript:

AACR	Animal-assisted crisis response
AAIs	Animal-assisted interventions
IRB	Institutional review board
