# Supplementary material for: The Human–Canine Partnership in Animal-Assisted Crisis Response: A Welfare-Centered Perspective
Source: Animals (Basel). 2026 Sep 11;16(18):2857. doi: 10.3390/ani16182857 (PMC13603382; doi:10.3390/ani16182857)
Supplement: Supplementary file 1 [file animals-16-02857-s001.zip › Supplementary Material S1. Semi-Structured Interview Guide (including the Participant Demo-graphic Questionnaire).pdf]

Interview Guides

Demographics for all the sample in separate online survey:

- Are you a licensed professional and if so, which?

- What is your role with Animal Assisted Crisis Response?

- (What is your role in the organization: AACR responder, Crisis Responder, Crisis Responder Supervisor)

- Gender

- Level religiosity/ religious practice: offer choices

- Do you come from a background where dogs are accepted?

- How long have you been doing Crisis Response work?

- How long have you been doing AACR?

Interview for AACR Responders

1. How long have you been involved with AACR?

2. Can you describe your training as an AACR Responder?

3. Can you describe the animal you are currently working with?

4. Do you also practice Crisis Response also without your therapy animal?

5. How does having a therapy animal affect your ability to do your job?

(What do you think happens differently when you have AACR on site in a crisis situation? For you? For the scene itself? What happens there?

6. From your experiences, how would you compare the trauma scene using AACR from not using AACR? (Does it change the interaction? In what ways does it change the interactions? Please explain.)

6.5 When using AACR have you noticed differences in the interactions between the survivors and the professionals at the scene?

6.6 Have you noticed any other differences you have not already mentioned?

7. For the first therapeutic contact, does AACR make a difference in the first contact with clients? If yes, in what ways?

8. Have you observed changes happening to the clients when having AACR at the scene? (What changes?)

9. Have you observed differences in clients' responses to AACR in comparison to CR without AACR? Please explain.

10. Do you find there are specific challenges related to arriving with a crisis support animal at a crisis scene? Please explain.

10.5 In terms of your relationship with your animal, are there challenges?

(Example: Is it hard for the pet? Does the animal obey your commands? Does the animal stress? etc.)

10.6 In terms of the relationships with the other professionals and the Crisis Response team at the crisis scene, can you describe their/ your reactions, and its effect, positive and negative, if any?

11. What works and what doesn't work with AACR?

11.5 In your view, what are the most important benefits of AACR?

11.6 Can you describe any problems you have noticed that can be attributed to the use of AACR?

12. Does having AACR as part of crisis response change the way you feel towards Crisis Response? If yes, please describe how.

13. Have you observed any changes in the way you engage in a crisis intervention? If yes, can you please describe how?

For questions 14 and 14.5, please consider the population characteristics such as age, gender, culture, and any other characteristics you can think of that is relevant to the questions.

14. From your experiences, are there specific populations that you see as benefitting most from AACR?

14. 5 Again from your experiences, are there groups that seem to benefit least from AACR?

15. Can you describe a crisis scene that having AACR resulted in a positive outcome?

16. Can you describe a crisis scene that having AACR may have contributed to problems at the crisis scene and your reactions?

17. Can you talk about a time that it would have been good to have AACR?

18. Overall, what is your view in the value of using AACR in a crisis scene?

19. On a scale from 1 to 5, when 1 is a terrible idea and 5 is an excellent idea, where would you put AACR?

Interview for mental health professionals Crisis Responders and work along AACR

1. How long have you been involved with AACR?

2. Tell me about your first experience with AARC?

3. How long have you been working along AACR?

4. How does it affect you in your job as Crisis Responder to work with AACR besides you?

5. From your experiences, how would you compare the trauma scene using AACR from not using AACR?

(Does it change the interaction? In what ways does it change the interactions? Please explain.)

5.5 When using AACR have you noticed differences in the interactions between the survivors and the professionals at the scene?

5.6 Have you noticed any other differences you have not already mentioned?

6. For the first therapeutic contact, does AACR make a difference in the first contact with clients? If yes, in what ways?

7. Have you observed changes happening to the clients when having AACR at the scene?

(What changes?)

8. Have you observed differences in clients' responses to AACR in comparison to CR without AACR? Please explain.

9. Do you find there are specific challenges related to arriving with a crisis support animal at a crisis scene? Please explain.

9.5 In terms of the relationships with the other professionals and the Crisis Response team at the crisis scene, can you describe their/ your reactions, and its effect, positive and negative, if any?

10. What works and what doesn't work with AACR?

10.5 In your view, what are the most important benefits of AACR?

10.6 Can you describe any problems you have noticed that can be attributed to the use of AACR?

11. Does having AACR as part of crisis response change the way you feel towards Crisis Response? If yes, please describe how.

For questions 12 and 12.5, please consider the population characteristics such as age, gender, culture, and any other characteristics you can think of that is relevant to the questions.

12. From your experiences, are there specific populations that you see as benefitting most from AACR?

12. 5 Again from your experiences, are there groups that seem to benefit least from AACR?

13. Can you describe a crisis scene that having AACR resulted in a positive outcome?

14. Can you describe a crisis scene that having AACR may have contributed to problems at the crisis scene and your reactions?

15. Can you talk about a time that it would have been good to have AACR?

16. Overall, what is your view in the value of using AACR in a crisis scene?

17. On a scale from 1 to 5, when 1 is a terrible idea and 5 is an excellent idea, where would you put AACR?

Interview for Supervisors of Mental health professionals Crisis Responders and AACR

1. How long have you been involved with AACR?

2. Tell me about your first experience with AARC?

3. How long have you been supervising AACR?

4. What criteria do you use to dispatch AACR to the scene?

5. From your experiences, how would you compare the trauma scene using AACR from not using AACR?

(Does it change the interaction? In what ways does it change the interactions? Please explain.)

5.5 When using AACR have you noticed differences in the interactions between the survivors and the professionals at the scene?

5.6 Have you noticed any other differences you have not already mentioned?

6. For the first therapeutic contact, does AACR make a difference in the first contact with clients? If yes, in what ways?

7. Have you observed changes happening to the clients when having AACR at the scene? (What changes?)

8. Have you observed differences in clients' responses to AACR in comparison to CR without AACR? Please explain.

9. Do you find there are specific challenges related to arriving with a crisis support animal at a crisis scene? Please explain.

9.5 In terms of the relationships with the other professionals and the Crisis Response team at the crisis scene, can you describe their/ your reactions, and its effect, positive and negative, if any?

10. What works and what doesn’t work with AACR?

10.5 In your view, what are the most important benefits of AACR?

10.6 Can you describe any problems you have noticed that can be attributed to the use of AACR?

11. Does having AACR as part of crisis response change the way you feel towards Crisis Response? If yes, please describe how.

For questions 12 and 12.5, please consider the population characteristics such as age, gender, culture, and any other characteristics you can think of that is relevant to the questions.

12. From your experiences, are there specific populations that you see as benefitting most from AACR?

12. 5 Again from your experiences, are there groups that seem to benefit least from AACR?

13. Can you describe a crisis scene that having AACR resulted in a positive outcome?

14. Can you describe a crisis scene that having AACR may have contributed to problems at the crisis scene and your reactions?

15. Can you talk about a time that it would have been good to have AACR?

16. Overall, what is your view in the value of using AACR in a crisis scene?

17. On a scale from 1 to 5, when 1 is a terrible idea and 5 is an excellent idea, where would you put AACR?
